# Why are the neurodegenerative disease-related pathways overrepresented in primary HIV-infected peripheral blood mononuclear cells: a genome-wide perspective

**DOI:** 10.1186/1743-422X-9-308

**Published:** 2012-12-16

**Authors:** Li Zhou, Viviane Conceicao, Priyanka Gupta, Nitin K Saksena

**Affiliations:** 1 Retroviral Genetics Division, Center for Virus Research, Westmead Millennium Institute, Westmead Hospital, the University of Sydney, Westmead, Sydney, 2145, NSW, Australia

**Keywords:** HIV-associated dementia, PBMCs, Neurodegenerative diseases, HIV, Microarray, Cell tagging

## Abstract

We demonstrate for the first time that the genome-wide profiling of HIV-infected peripheral blood mononuclear cells (PBMCs) from HIV-patients free of neurologic disease show overrepresentation of neurodegenerative pathways (Alzheimer’s, Parkinson’s, ALS, Huntington’s and Prion Disease, etc.) in genome-wide microarray analysis, which suggests that this genome-wide representation of neurodegenerative diseases-related pathways in PBMCs could possibly be a subcellular manifestation of neurologic interference by HIV. Further, the cell-tagging analysis attested this belief showing the large majority of genes tagged with cells of monocyte and macrophage lineage, which are implicated in neuronal dysfunction in both viral and non-viral neurodegenerative diseases. Together, these findings suggest that the genomic interference of HIV with neurodegenerative pathways is not by chance, but may be an early sign of HIV-mediated sub-genomic and sub-cellular manifestation of neurologic disease. Moreover, these findings signify the utility of PBMC and genome-wide mapping of the host gene expression as a powerful tool in predicting possible early events in neurologic deterioration in HIV patients.

## Findings

Neurological impairment is a common feature of Acquired Immunodeficiency Syndrome (AIDS) and other virus-mediated neurodegenerative diseases. Functional alterations occur in both in central and peripheral nervous system. Despite the success of highly active antiretroviral therapy (HAART) at reducing the incidence of HIV-associated neurocognitive disorders (HAND), there are still nearly 50% HIV-infected individuals who are predisposed to multiple cognitive domain deficits, such as psychomotor slowing, attention, memory, working memory, executive function, abstraction, verbal fluency, speed of information processing, sensory perceptual, and motor speed [1], which eventually will translate into HIV-associated dementia in >25% of HIV+ individuals on HAART. So far, HAD remains to be one of the most devastating complications of HIV infection, which significantly interferes with the quality of life of HIV+ individuals.

Although extensive research has been carried out to define the onset, progression and prognosis of HAND, to date the reflection of neurologic predisposition has not been looked at systemically, especially at the level of whole cellular transcriptome. In the recent years, gene expression profiling has become one of the key approaches to mining the differentially expressed genes between disease and control groups, which is rapidly changing the landscape of diagnostics and prognostics in general. It has also added another dimension to the way we look at and treat different diseases. It is important to reiterate that brain biopsy is not a clinically feasible option and it is severely restricted in the clinical context. In these cases, peripheral blood mononuclear cells (PBMCs), which are involved in numerous immune related diseases, serve as highly valuable and informative surrogate material for gene expression studies. Recently, it has been shown by several studies that gene expression in PBMCs is altered in the context of cancer [2], sepsis [3], asthma [4], familial hemophagocytic lymphohistiocytosis [5], and endometriosis [6]. This information can translate into clinically beneficial outcomes both at the treatment and diagnostic fronts. Moreover, it has also been shown that increased caspase activation and deregulation of stress genes in PBMCs of patients with Alzheimer's disease [7,8]. Thus, the PBMCs are widely regarded as an attractive component in diagnosis and prognosis of a variety of diseases.

Here, we have carried out a genome-wide profiling of the PBMCs from healthy controls and HIV-infected patients. 5 subjects from each group have been used. All the subjects are male, average age for healthy controls is 47.5 ± 3.5 and 50 ± 6.67 for HIV infected patients. For HIV infected patients, the average duration for HIV infection is 13.4 years ± 3.51; the pre-therapy viral load varies from 5208 to 750,000, the average is 349301.6; CD4+ T cell count is from 360 to 1050, the average is 658; CD8+ T cell count is from 680 to 1380, the average is 1025.8. Extensive clinical neurocognitive examination has been carried out on all the subjects and they are free of any form of HIV-associated neurologic disease according to the current diagnostic criteria for HAND defined by the American Academy of Neurology 2007 [9]. We compared the PBMC profiling results with our genome-wide profiling of the autopsied HIV-infected brains with and without neurological disorders. 10 HAND patients and 7 HIV patients with no neurological disorder were used for genome-wide profiling. All of them are male, except one. The average age is 47.5±12.85. The duration of HIV seropositivity to death is from 9 months to 22 years. None of the HAND patients have any non-HAND neuropathology, while only one HIV non-neurological disorder patient showed minimal non-diagnostic abnormalities. (This study was conducted according to the principles expressed in the Declaration of Helsinki. Use of HIV positive and negative peripheral blood samples in this study was approved by the Institutional Review Board and the Ethics Committee of the University of Sydney and the Royal Prince Alfred Hospital, Sydney and Westmead Hospital (Ref: HREC2008/6/4.10 (2817). The patients or family members of the patients gave written, informed consent for the use of blood sample and autopsied brain tissue. The brain autopsied tissue samples, obtained from the Mount Sinai School of Medicine, were covered the Human Ethics of the institution and also under the Anatomical Gift Program of the Neuropathology Research Division and The Manhattan HIV Brain Bank, N.Y. USA. In each case appropriate consent was obtained from the family of deceased who participated in donating brain tissue for research (Study ID# 98-477 (0002)PA).

**Figure 1:**
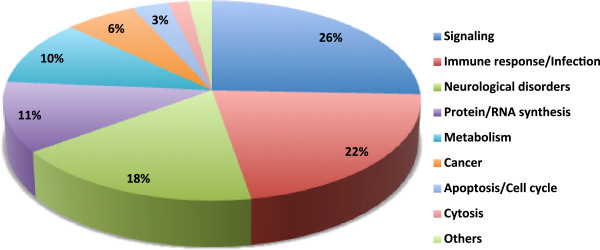
**Representation of statistically significant enriched pathways of PBMC mRNA profiles.** Pie chart representing pathway analysis. Each part of the pie chart represents -log_2_ of the P-value of pathway from the set of significant pathways, where the total of -log_2_ of the P-value is 1. The P-values were retrieved from the pathway analysis in WebGestalt.

**Figure 2:**
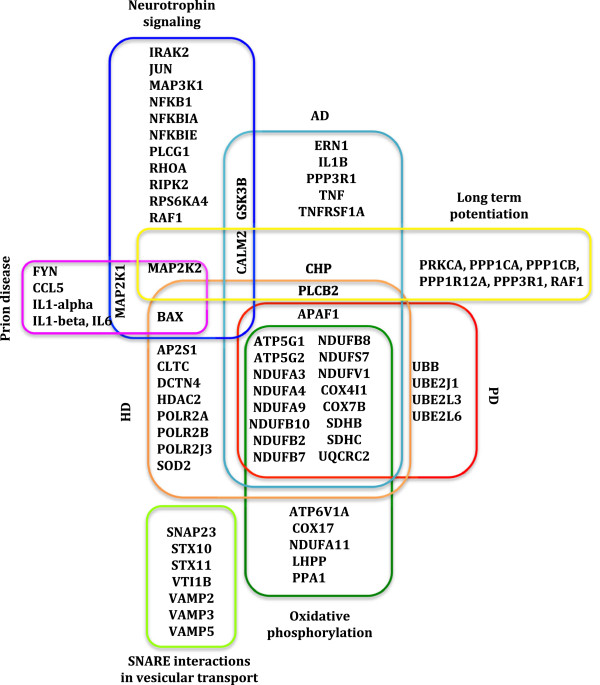
**Illustration of altered DE genes in neurological disease-related pathways.** Representation of DE genes between PBMCs from HIV positive individuals and healthy controls, which fell into statistically significant enriched neurological disease-related pathways. Different colors have been adopted to highlight DE genes in individual pathways.

Transcriptomic profiling of PBMCs revealed 1089 genes significantly dysregulated (P<0.05), among them 491 were up regulated and 599 down regulated (Additional file 1). The differentially expressed genes were uploaded to the WEB-based GEne SeT AnaLysis Toolkit (WebGestalt) to explore functionally relevant pathways. We found significant involvement of signaling (25%), immune response/infection (22%), neurological disorders (18%), and metabolism (11%) (Figure 1), which is highly consistent with our previous genomic and proteomic studies carried out using brain tissue from HIV-infected patients with and without neurological disorders (Additional file 2) [10]. Not surprisingly, mainly the immune response/infection related pathways, such as immunodeficiency, cytokine and their receptor interaction, IgA production, and antigen presentation were significantly enriched in PBMC-derived from HIV+ patients. In addition, we also found significant enrichment of signaling pathways, including MAPK signaling, TGF-beta signaling and Neurotrophin signaling pathways, etc. But the most notable was the significant enrichment of neurological disease-related pathways (Figure 2) comprising of Alzheimer disease (AD) (P=2.88E-11), Parkinson’s disease (PD) (P=1.25E-11), Huntington’s disease (P=7.03E-9) (HD), Prion diseases (P=5.31E-6), etc. This striking overrepresentation of neurodegenerative pathways in HIV-infected PBMC at the genomic level as well its concordance with our recent neuro-proteomic study has considerable significance in explaining early systemic interference with host neurological function by HIV, which genetically predisposes HIV patients to neurologic disease. In this context, it is important to reiterate some studies have found that within the first year of HIV infection, there is significantly reduced co-activation within lateral occipital cortex network compared to healthy control, which correlates with the performance on tasks involving visual-motor coordination [11]. Functional magnetic resonance imaging (fMRI) has also shown that HIV positive individuals have a lower baseline cerebral blood flow and was equivalent to a 21 year increase in brain age compared to HIV negative controls. It is important to note that HIV-infected individuals in some cases develop AD and PD, but at a much younger age compared to HIV negative AD and PD patients. Also, in other cases HIV patients also display cognitive difficulties and behavioral abnormalities including psychosis and depression [12,13]. Furthermore, HAD patients share common anatomical substrates (hippocampus and substantia nigra) [14] and pathological biochemical change (dopamine deficiency and testosterone deficiency) with AD and PD. Therefore, our findings on genomic interference of HIV with neurodegenerative pathways are not by chance, but they are possibly the early hints of HIV interference with host neurological function and possible indicator of sub-cellular or sub-genomic manifestation of neurologic disease.

**Figure 3:**
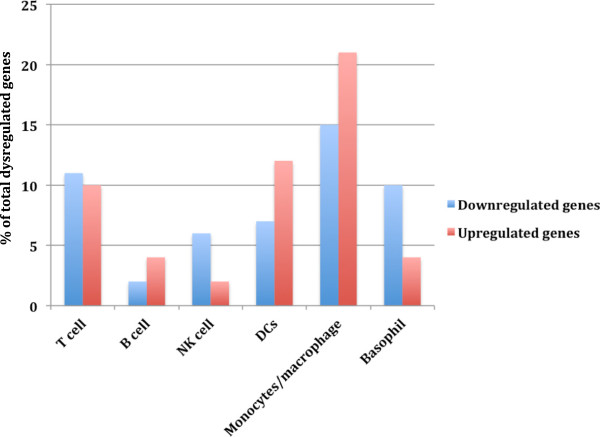
**Cell-tagging analysis.** Diverse cell types in PBMCs tagged by dysregulated genes. Percentage of total dysregulated genes (P<0.05) in HIV positive PBMCs versus healthy controls, expressed in T-cells, B-cells, natural killer (NK)-cells, monocytes/macrophage, dendritic cells (DCs), and basophils, determined from the human immune cell module of the immunological genome project [15]. A much higher percentage of the DE genes were predominantly expressed in monocytes/macrophage and dendritic cells (DCs), consistent with HIV infection of the brain. There was no significant difference between up- and down-regulated genes. P-values were determined by comparing the number of genes from each cell subset observed and expected in either up or down regulated genes.

To further point out at the specific cell subset(s) responsible for this functional enrichment of genes in the neurodegenerative disease-related pathways, cell subset tagging was carried out using immunological genome website [15]. Dysregulated genes between PBMCs from HIV+ individuals and healthy controls, which showed significant involvement in neurological diseases related pathways, were uploaded to this website. Genes, predominantly expressed in one cell subset, were identified and the percentage of genes tagging the cell subsets from our uploaded DE genes is shown in Figure 3 and Table 1. Various T lymphocytes tagging genes were found involved in the HD pathway, which is consistent with previous findings of T-lymphocyte impairment in HD [16]. Most interestingly, >50% of the DE genes contributing to neurological diseases pathways tagged largely with cells of monocyte/macrophage lineage (monocytes, macrophage and dendritic cells) (P<0.05) shown to play a crucial role in the pathogenesis of HAD. These results are consistent with our unpublished data (Table 2). These results also concur with the findings that monocytes are a critical cellular subset as HIV DNA copies in monocytes correlate with cognitive performance and can be used to predict 48-week cognitive performance [17], although previous studies reported HIV DNA in PBMCs are proportionally correlative to the severity of HAND regardless of HIV RNA levels in the plasma [18]. Furthermore, HIV DNA levels in PBMCs are even associated with individual deficits in neurocognitive domains [18-21]. These results also provide further support to the evidence that HAND might be triggered by HIV-infected/activated M/Mϕ in the peripheral system, which bears a strong correlation with HAND severity [22]. Supporting these observations, increased monocytes trafficking into the brain has also been found in inflammatory diseases occurring outside the central nervous system (CNS) [23]. Besides, using genetically- labelled bone marrow-derived cells, evidence has also shown enhanced engraftment of labeled monocytes and their differentiation to microglia during the post-inflammatory period, and most importantly the integration of these newly recruited monocytes into the pool of parenchymal microglia [24], further unifying the role of systemic monocyte/Mϕ cell lineages in the manifestation of HAD.

**Table 1:** Gene expression in diverse blood leukocytes using cell tagging

**GENE SYMBOL**	**T cell**	**B cell**	**NK cell**	**DCs**	**Monocytes/macrophage**	**Basophil**
AP2S1						*
APAF1	*					*
ATP5G1				*		
ATP5G2	*	*				
ATP6V1A					*	
BAX	*					
CALM1	*					*
CCL5					*	
CHP					*	
CLTC				*	*	
COX17						*
COX4I1				*		
COX7B					*	*
DCTN4				*	*	
ERN1	*					
FYN		*				
GSK3B					*	*
HDAC2	*			*	*	
IL1-alpha					*	
IL1-beta					*	
IL6					*	
IRAK2						
JUN					*	
LHPP		*				
MAP2K1	*			*	*	
MAP2K2	*		*			
MAP3K1	*		*		*	
NDUFA11						
NDUFA3						*
NDUFA4						*
NDUFA9				*		
NDUFB10	*					
NDUFB2					*	
NDUFB7					*	
NDUFB8	*			*	*	
NDUFS7				*	*	
NDUFV1	*	*		*		
NFKB1					*	
NFKBIA				*	*	
NFKBIE				*		
PLCB2				*		
PLCG1	*					
POLR2A	*		*			*
POLR2B						*
POLR2J2	*					
PPA1	*					
PPP1CA	*					
PPP1CB	*				*	
PPP1R12A						*
PPP3R1					*	
PRKCA				*		
RAF1					*	
RHOA		*	*		*	*
RIPK2					*	
RPS6KA4			*			
SDHB				*	*	
SDHC					*	
SNAP23						*
SOD2					*	
STX10	*			*	*	
STX11					*	
TNF					*	
TNFRSF1A			*	*	*	
UBB			*		*	
UBE2J1		*				
UBE2L3	*					
UBE2L6				*		
UQCRC2	*				*	
VAMP2			*			
VAMP3					*	
VAMP5				*		
VTI1B					*	*

NOTE: *indicates the highest expression cellular subtype.

**Table 2:** Enriched KEGG pathways derived from differentially expressed genes in monocytes in comparison to viremic patients versus long term non-progressors

**Enriched pathway**	**Enrichment statistics**
**Cell signaling**	
***Chemokine signaling pathway***	C=190;O=15;E=1.12;R=13.41;rawP=6.29e-13;adjP=2.64e-11
***Toll-like receptor signaling pathway***	C=101;O=11;E=0.59;R=18.49;rawP=2.39e-11;adjP=5.02e-10
***NOD-like receptor signaling pathway***	C=62;O=9;E=0.37;R=24.65;rawP=1.15e-10;adjP=1.61e-09
Neurotrophin signaling pathway	C=126;O=10;E=0.74;R=13.48;rawP=4.48e-09;adjP=4.70e-08
***Cytokine-cytokine receptor interaction***	C=267;O=13;E=1.57;R=8.27;rawP=8.49e-09;adjP=7.13e-08
T cell receptor signaling pathway	C=108;O=7;E=0.64;R=11.01;rawP=3.81e-06;adjP=1.78e-05
***MAPK signaling pathway***	C=269;O=10;E=1.58;R=6.31;rawP=5.15e-06;adjP=1.97e-05
Epithelial cell signaling in Helicobacter pylori infection	C=68;O=5;E=0.40;R=12.49;rawP=5.25e-05;adjP=0.0001
B cell receptor signaling pathway	C=75;O=5;E=0.44;R=11.32;rawP=8.41e-05;adjP=0.0002
VEGF signaling pathway	C=76;O=5;E=0.45;R=11.17;rawP=8.96e-05;adjP=0.0002
ErbB signaling pathway	C=87;O=4;E=0.51;R=7.81;rawP=0.0018;adjP=0.0023
Insulin signaling pathway	C=137;O=4;E=0.81;R=4.96;rawP=0.0090;adjP=0.0095
Wnt signaling pathway	C=151;O=4;E=0.89;R=4.50;rawP=0.0125;adjP=0.0128
Jak-STAT signaling pathway	C=155;O=4;E=0.91;R=4.38;rawP=0.0136;adjP=0.0136
**Cell cycle and apoptosis**	
Lysosome	C=117;O=7;E=0.69;R=10.16;rawP=6.49e-06;adjP=2.27e-05
Apoptosis	C=88;O=6;E=0.52;R=11.58;rawP=1.43e-05;adjP=4.62e-05
Cell cycle	C=128;O=4;E=0.75;R=5.31;rawP=0.0071;adjP=0.0081
**Cytoskeleton and cell migration**	
***Leukocyte transendothelial migration***	C=118;O=5;E=0.69;R=7.20;rawP=0.0007;adjP=0.0012
Regulation of actin cytoskeleton	C=216;O=6;E=1.27;R=4.72;rawP=0.0019;adjP=0.0023
Focal adhesion	C=201;O=5;E=1.18;R=4.22;rawP=0.0070;adjP=0.0081
**Phagocytosis**	
Endocytosis	C=187;O=6;E=1.10;R=5.45;rawP=0.0009;adjP=0.0014
Fc gamma R-mediated phagocytosis	C=97;O=4;E=0.57;R=7.00;rawP=0.0027;adjP=0.0032
Natural killer cell mediated cytotoxicity	C=137;O=4;E=0.81;R=4.96;rawP=0.0090;adjP=0.0095
**Metabolism**	
Glycolysis/Gluconeogenesis	C=62;O=7;E=0.37;R=19.17;rawP=8.47e-08;adjP=5.93e-07
Metabolic pathways	C=1104;O=18;E=6.50;R=2.77;rawP=0.0001;adjP=0.0002
Oxidative phosphorylation	C=135;O=4;E=0.80;R=5.03;rawP=0.0085;adjP=0.0094
**Miscellaneous**	
Prion diseases	C=35;O=5;E=0.21;R=24.26;rawP=1.92e-06;adjP=1.01e-05
Bladder cancer	C=42;O=5;E=0.25;R=20.22;rawP=4.86e-06;adjP=1.97e-05
Long-term depression	C=70;O=5;E=0.41;R=12.13;rawP=6.04e-05;adjP=0.0002
Endometrial cancer	C=52;O=4;E=0.31;R=13.06;rawP=0.0003;adjP=0.0006
Oocyte meiosis	C=114;O=5;E=0.67;R=7.45;rawP=0.0006;adjP=0.0010
Glioma	C=65;O=4;E=0.38;R=10.45;rawP=0.0006;adjP=0.0010
Alzheimer's disease	C=169;O=6;E=1.00;R=6.03;rawP=0.0005;adjP=0.0010
Melanoma	C=71;O=4;E=0.42;R=9.57;rawP=0.0008;adjP=0.0013
Axon guidance	C=129;O=5;E=0.76;R=6.58;rawP=0.0010;adjP=0.0015
Tight junction	C=134;O=5;E=0.79;R=6.34;rawP=0.0012;adjP=0.0017

Overall, the data presented herein leads us to believe that the intrinsic signatures of neurodegenerative pathways we show in the systemic circulation are not there by chance and may have vital functional significance as early indicators of HIV interference with host neurological functions at the sub-genomic or sub-cellular level, which possibly occurs far ahead of actual clinical manifestation and outside of CNS. Any effective interference at an early stage and targeting peripheral blood or even particular cell subsets should promise a better disease prognosis or even prevention of neurological slide in HIV patients. If attended later, some pathological changes might become irreversible and may manifest for life compromising the blood brain barrier and drug penetration. Together, these studies provide a new paradigm in early neurological interference by HIV and carry immense significance in early prevention and drug intervention towards neurologic deterioration in HIV-infected patients.

## Conclusions

In summary, we provide a unique viewpoint that raises important questions about transcriptomic expression in HIV-infected PBMC of HIV patients without neurologic disease and its possible role as an early indicator of genomic programming and sub-genomic manifestation of HIV neurologic disease. Since the large majority of genes implicated in neurodegenerative diseases tagged with cells of monocyte and macrophage lineage, which play a crucial role in a variety of NDs, it is plausible to hypothesize that these cells by infiltrating across the blood brain barrier can carry out early programming of events in the CNS leading to neurological manifestation. Overall, these findings suggest possible sub-genomic/sub-cellular neurological deficit at an early stage of HIV infection with significant contribution of M/Mϕ lineages.

## Competing interests

The authors declare that they have no competing interests.

## Authors’ contribution

LZ fully drafted the short report. VC and PG carried out part of the technical work. NKS conceived, designed, supervised and coordinated the study, along with providing assistance with drafting the manuscript. All authors read and approved the final manuscript.

## Supplementary Material

Additional file 1 Significantly differentially expressed gene between the PBMCs from healthy controls and HIV-infected patients. Click here for file

Additional file 2 Representation of gene ontology biological processes of mRNA profiles. Click here for file
