# Supplementary material for: Why are the neurodegenerative disease-related pathways overrepresented in primary HIV-infected peripheral blood mononuclear cells: a genome-wide perspective
Source: Virol J. 2012 Dec 16;9:308. doi: 10.1186/1743-422X-9-308 (PMC3546955; doi:10.1186/1743-422X-9-308)
Supplement: Additional file 2 — Representation of gene ontology biological processes of mRNA profiles. [file 1743-422X-9-308-S2.pptx]

## Slide 1
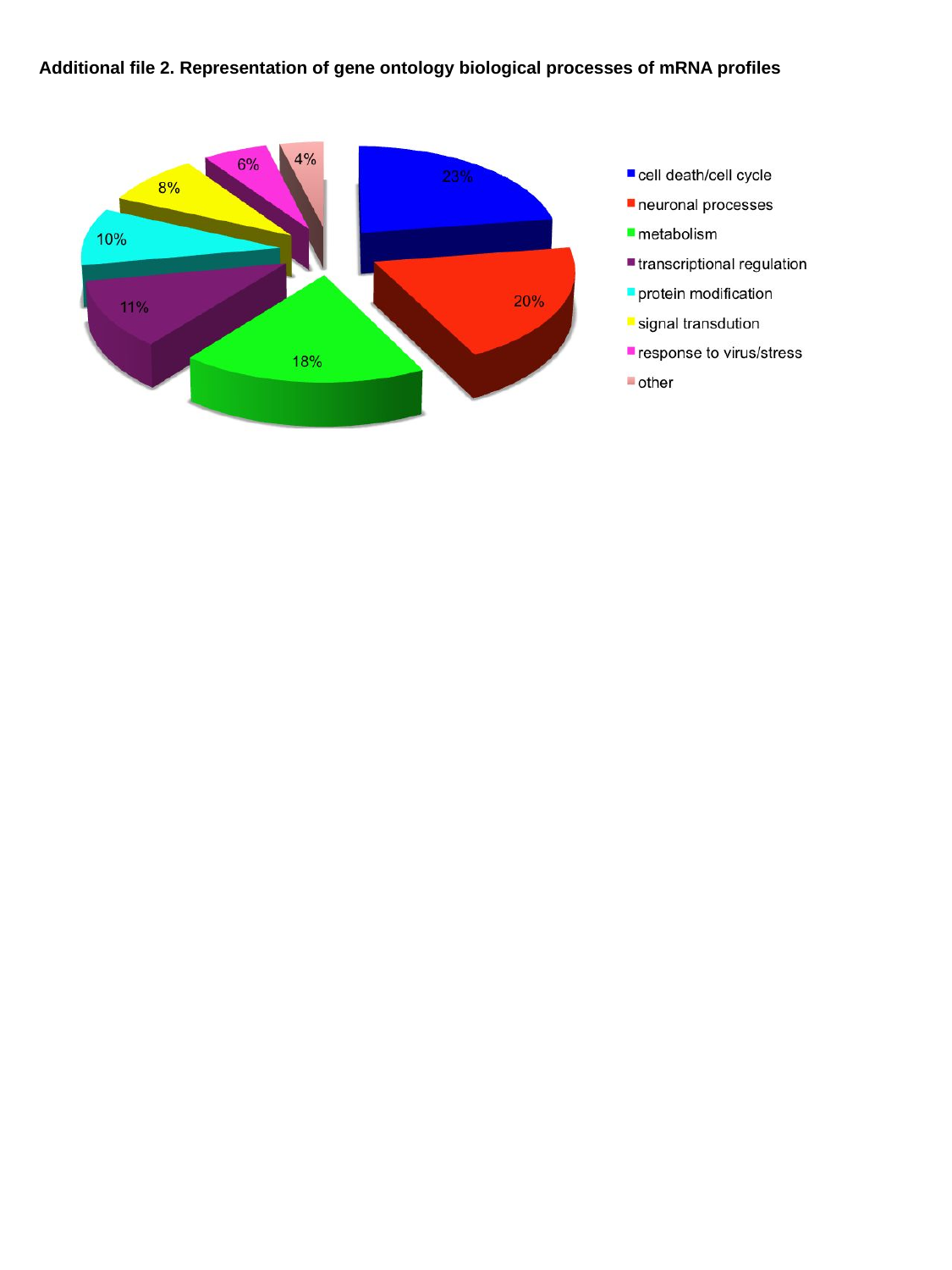

Additional file 2. Representation of gene ontology biological processes of mRNA profiles
